# Evaluation of time to positivity for blood culture combined with immature granulocytes, neutrophil‐to‐lymphocyte ratio, and CRP in identifying bloodstream coagulase‐negative *Staphylococci* infection in pediatric patients

**DOI:** 10.1002/jcla.23473

**Published:** 2020-07-19

**Authors:** Lanlan Zeng, Shuhui Wang, Minchun Lin, Yaxing Chen, Qiulian Deng, Huamin Zhong, Xiaoshan Guan, Shuwen Yao, Haiying Liu

**Affiliations:** ^1^ Guangzhou Women and Children's Medical Center Guangzhou Medical University Guangzhou China

**Keywords:** bloodstream infection, coagulase‐negative *staphylococcus*, immature granulocytes, pediatric patients, time to positivity

## Abstract

**Objective:**

To evaluate the application value of time to positivity (TTP) for blood culture combined with inflammatory parameters that included immature granulocyte percentage (IG%), immature granulocyte count (IG#), C‐reactive protein (CRP), white blood cells (WBC) neutrophil percentage (NE%), and neutrophil‐to‐lymphocyte ratio (NLR), and to identify bloodstream infections from contamination with coagulase‐negative *staphylococci* (CoNS) in pediatric patients.

**Methods:**

Data of 12 897 inpatients with blood culture CoNS were retrospectively collected and analyzed from January‐December 2019 at our hospital. According to pre‐defined criteria, they were divided into a CoNS infection group (132 cases) and a CoNS contamination group (124 cases). Infection with *Staphylococcus aureus* (SA, 27 cases) at the same period was considered a positive control group. ROC curve analysis assisted in determining the value of applying TTP combined with the above‐mentioned inflammatory parameters to distinguish CoNS infection from contamination.

**Results:**

Among the 256 strains of CoNS, *Staphylococcus hominis* (55.1%), *Staphylococcus epidermidis* (32.0%), and *Staphylococcus capitis* (7.0%) were common. There was no significant difference in the subspecies distribution between the infection and contamination groups. The TTP of the CoNS infection group was significantly lower than the contamination group (*P* < .05). IG%, IG#, CRP, NE%, and NLR were all higher in the infected group as compared to the contaminated group (*P* < .05), while WBC was similar among groups. There was also no statistical difference in those parameters when comparing the CoNS infection and SA groups. ROC analysis showed that TTP value in identifying CoNS infection from contamination was the highest with area under the curve (AUC) of 0.913, and the sensitivity and specificity were 0.827 and 0.852, respectively, at the optimal cutoff value of 23.9 hours. This was followed by IG% (AUC = 0.712), with an optimal critical value of 0.55%, and a sensitivity of 0.519 and specificity of 0.797. All the AUC values of IG#, CRP, NE%, and NLR were <0.7. A combination of TTP with IG%, CRP, and NLR improved the AUC, sensitivity, specificity, accuracy, PPV, and NPV values to 0.977, 0.922, 0.957, 91.8%, 92.2%, and 91.3%, respectively.

**Conclusions:**

TTP within 24 hours indicates likelihood of CoNS as the pathogenic agent in pediatric patient blood culture. The combination of TTP with IG% CRP and NLR might improve the diagnostic accuracy.

## Graphical abstract

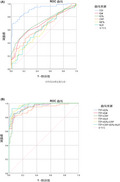

## 1 INTRODUCTION

Coagulase‐negative *Staphylococci* (CoNS) including *Staphylococcus epidermidis, Staphylococcus hominis*, and *Staphylococcus capitis* among others, which cannot produce substances like plasma coagulase and hemolysin, are considered normal flora or opportunistic pathogens of the skin mucosa. In recent years, with the extensive use of biomedical devices and the widespread application of various broad‐spectrum antibiotics, CoNS has emerged as an important pathogen of hospital infection and the most common bacteria that causes blood culture contamination.^1^ Blood culture is the gold standard for bloodstream infections.

As a common parameter in blood culture, the clinical predictive value of time to positivity (TTP) on bloodstream infection has also attracted increased attention.^2^, ^3^ TTP refers to a period of time from the moment the blood is isolated from the body to the positive alarm of the blood culture instrument. This is related to the initial concentration of bacteria, and can be used to differentiate the diagnosis of whether a blood culture of CoNS is an infection or due to contamination.

Immature granulocyte IG is a new detection index for the diagnosis of infectious diseases, including promyelocytic, and middle and late granulocytes, which belong to the precursor stage of developing mature white blood cells. Immature granulocytes generally do not exist in the peripheral blood of healthy people; however, severe clinical conditions (such as sepsis and septicemia) can consume a large number of peripheral blood granulocytes, and the body compensates by releasing immature granulocytes from the bone marrow to the peripheral blood circulation. Therefore, peripheral immature granulocytes can be regarded as a new indicator to reflect the severity of inflammation.

Peripheral blood neutrophil‐to‐lymphocyte ratio as an indicator reflecting the body's systemic inflammatory response and immune status has attracted widespread attention in recent years. According to reports, it has proven its accuracy in predicting the outcome of patients with major cardiac events, ischemic stroke, cancers, sepsis, and infectious pathologies.^4^ The mark is simple, easy to obtain and calculate, and easy to integrate into daily practice without paying extra.

In this current study, clinical data and laboratory inflammatory indicators of the included children were retrospectively analyzed to explore the value of blood culture positive alarm time TTP when combined with common inflammatory indicators (eg, WBC and CRP), including IG, NLR in the differential diagnosis of bloodstream infections, and contamination.

## 2 MATERIALS AND METHODS

### 2.1 Patient population

A total of 256 coagulase‐negative staphylococci positive cases with complete case data were selected from the blood culture records of 12 897 inpatients of children in our hospital from January‐December 2019. Moreover, 27 cases of coagulase‐positive *Staphylococcus aureus* in the same period were collected as positive controls. Inclusion criteria included the following: (a) From the blood culture specimens, first time detection of the staphylococcus genus; (b) no other bacteria or fungi isolated from the blood cultured specimens of this patient; and (c) specimens submitted by one person for multiple tests were only included in the results of the first test. The TTP of coagulase‐negative staphylococci and their corresponding bacterial species in the blood culture of patients were recorded, and their clinical data were collected. This included the clinical diagnosis, clinical manifestations, basic disease characterization, use of an indwelling catheter, body temperature at the time of blood sample extraction, and various indicators of infection in the laboratory testing analyses (including CRP, WBC, IG%, IG#, NE%, and NLR), application of antibiotics, and therapeutic effect. All data were divided into a CoNS infection group, a CoNS contamination group, and an SA control group according to the grouping criteria.

### 2.2 Criteria for judging CoNS of bloodstream infections and contamination

According to the definition of nosocomial infection surveillance published by the US^5^ Centers for Disease Control and Prevention (CDC) in 2016, at least one of the following one criteria are met: ① one or more sets of blood culture or identification of the CoNS pathogen through a non‐culture microbiological detection assay, and blood microorganisms and other site infections being irrelevant; ② the patient had at least one of the following symptoms or signs: a body temperature of >38°C, chills, hypotension, and evidence that the same CoNS were isolated from blood culture on two or more occasions under different circumstances, and the above standard elements must occur in the window period of infection; that is, the day of positive blood sampling or 3 days before and after the window of infection; and ③ patients with an age of less than or equal to 1 years old, having at least one of the following symptoms or signs: evidence of fever (>38°C), or a low temperature (<37°C), apnea, bradycardia, and at least two positive blood cultures that detected the same CoNS. The detected microorganisms must also be confirmed not to be related to other site infections. According to this standard, the selected cases were divided into two groups, namely, the infection group and the contamination group.

### 2.3 Experimental method

Specimen collection: in the early stage of fever or chills, samples should be collected before antibiotic treatment, and blood samples should be collected in strict accord to the aseptic procedures before blood drawing occurs. The amount of blood taken from children should be approximately 1‐3 mL. After the blood is drawn, it should be immediately injected into a culture bottle especially designed for children (BD Company). After receiving the samples and being scanned and recognized by the instrument, the samples were directly placed into the blood culture instrument and incubated for 7 days. The instrument observed and recorded the growth curve and the positive alarm time in real‐time. Meanwhile, 2 mL of venous blood was extracted and mixed in an EDTA‐k2 anti‐coagulation tube.

Treatment of positive culture bottle: the BACTECTM‐FX automated blood culture instrument (BD Company, USA) was used. When the instrument indicated a positive alarm, blood plate and chocolate plate were transferred in a timely manner and cultured in a 5% CO_2_ incubator for 18‐24 hours to separate a single colony. A Vetak‐2 automatic microbial identification instrument (Biomerere, France) was used for pathogen identification.

Specimen detection: the USES xn‐1000 automatic hematological analyzer (Japan Sysmex Company) was used, and reagents were obtained from the original package of supporting reagents. IG was detected by sheath flow technology, electrical impedance technology, and nucleic acid fluorescence staining. In addition, relevant parameters such as IG percentage (IG%), IG count (IG#), total white blood cell (WBC) counts and classification, neutrophil‐to‐lymphocyte ratio (NLR) were recorded.

Reference range values: IG% 0%‐0.35%; IG# 0‐0.04 × 10^9^/L; Where IG% = IG#/WBC count × 100. (2)

CRP detection: the instrument adopted the OTTOMAN ‐ 1000 special protein analyzer that is available from the Shanghai Opu Bio‐Pharmaceutical Co., Ltd., and detected by the latex enhanced transmission immuno‐turbidimetric method, with a reference range of 0‐8 mg/L.

### 2.4 Statistical analysis

The SPSS version 26.0 statistical software program was used to process the data. The Shapiro‐Wilk W test showed that age, TTP, IG#, IG%, CRP, WBC, NE%, and NLR all showed a skewed distribution, and the data were represented by the median (interquartile range) [M (P25, P75)]. The Mann‐Whitney U rank sum test was used for inter‐group comparisons. The data of each parameter are expressed by the number of examples or percentage values. The sample rate was compared by the chi‐square test. The receiver operating characteristic (ROC) curve was plotted, and the optimal critical value was determined. The area under the curve (AUC), sensitivity, specificity, positive predictive value, negative predictive value, and accuracy were all calculated. An AUC > 0.9 has a highly prompt accuracy, a value of 0.7‐0.9 has a medium prompt accuracy, and a value of 0.5‐0.7 has low prompt accuracy. An alpha value of *P* < .05 for differences between data groups is considered statistically significant.

## 3 RESULTS

### 3.1 Clinical data

A total of 283 cases were included in the study, including 197 males and 86 females. According to the grouping criteria, 132 cases were included in the CoNS infection group, 124 cases in the contamination group, and 27 cases in the *Staphylococcus aureus* (SA) control group. The age of the infection group was 1.74 (0.45, 5.01) years old, that of the contamination group was 2.08 (0.47, 4.90) years old, and that of the SA group was 1.3 (0.4, 8.00) years old. There was no statistical difference in the context of gender and age among any of the three groups.

### 3.2 Distribution of CoNS species in the infection and contamination groups

A total of 256 CoNS strains were isolated from 12,897 cases (see Table 1), among which the proportion of human *Staphylococcus* was the highest, with a total composition ratio of 55.1%. The others were *Staphylococcus epidermis* (32.0%), *Staphylococcus cephalus* (7.0%), *Staphylococcus haemolyticus* (2.3%), and *Staphylococcus goat* (1.2%). There was no statistical difference in the distribution composition ratio of CoNS between the infection and the contamination groups (*P* > .05).

**TABLE 1:** Distribution of CoNS subspecies in groups with bloodstream infection and contamination

CoNS	Infection group		Contamination group		Total	
	Number	Composition ratio	Number	Composition ratio	Number	Composition ratio
Staphylococcus hominis	76	57.6%	65	52.4%	141	55.1%
Staphylococcus epidermidis	38	28.8%	44	35.5%	82	32.0%
Staphylococcus capitis	10	7.6%	8	6.5%	18	7.0%
Staphylococcus haemolyticus	4	3.0%	2	1.6%	6	2.3%
Staphylococcus caprae	2	1.5%	2	1.6%	3	1.2%
Other Staphylococcus	2	1.5%	3	2.4%	6	2.4%
Total	132	100%	124	100%	256	100%

### 3.3 Comparison of TTP and inflammatory parameters among different groups

In Table 2, the TTP time of the CoNS infection group was significantly earlier than that of the contamination group [22.4(17.6, 31.1)h vs 31.1(25.3, 41.3)h, *P* < .05], and IG% [0.5(0.3, 0.8) vs 0.3(0.2, 0.5)], IG# [0.04 (0.02, 0.09) × 10^9^/L vs 0.03(0.02, 0.05) × 10^9^/L], CRP[9.0 (1.1, 32.1)mg/L vs 4.2 (0.6, 15.4)mg/L], NE% [48 (32.8, 65.0) vs 44 (28.5, 59.5)], and NLR[1.56(0.76, 3.23) vs 0.96(0.455, 2.21)] which were all higher than that of the contamination group (*P* < .05). There was no significant difference in WBC between groups (*P* > .05). There was no statistically significant difference between indicators of the CoNS infection and the SA groups (*P* > .05; Table 2).

**TABLE 2:** Comparison of TTP and inflammatory parameters among different groups [M (P25, P75)]

Group	n	TTP (h)	CRP (mg/L)	WBC (×10^9^/L)	IG (%)	IG# (×10^9^/L)	NE (%)	NLR
Infection group	132	22.4 (17.6,31.1)	9.0 (1.1,32.1)	10.1 (6.9,13.3)	0.5 (0.3,0.8)	0.04 (0.02,0.09)	48 (32.8,65.0)	1.56 (0.76,3.23)
Contamination group	124	31.1 (25.3,41.3)	4.2 (0.6,15.4)	9.2 (7.0,11.6)	0.3 (0.2,0.5)	0.03 (0.02,0.05)	44 (28.5,59.5)	0.96 (0.455,2.21)
SA group	27	16.4 (11.3,26.1)	23.84 (3.68,79.1)	11.2 (7.0,13.6)	0.55 (0.5,1.1)	0.08 (0.05,0.16)	58.5 (40.8,70.0)	1.74 (1.36,3.02)
*U* *	/	496.0	1964.0	2907.5	1603.5	1767.0	2445.0	2193
*P* *	/	.000	.001	.645	.000	.000	.036	.029

^*^ Note: comparison between the CoNS infection group and the contamination group.

### 3.4 ROC analysis and efficacy evaluation of TTP and inflammatory parameters in identifying CoNS bloodstream infection from contamination

**FIGURE 1:**
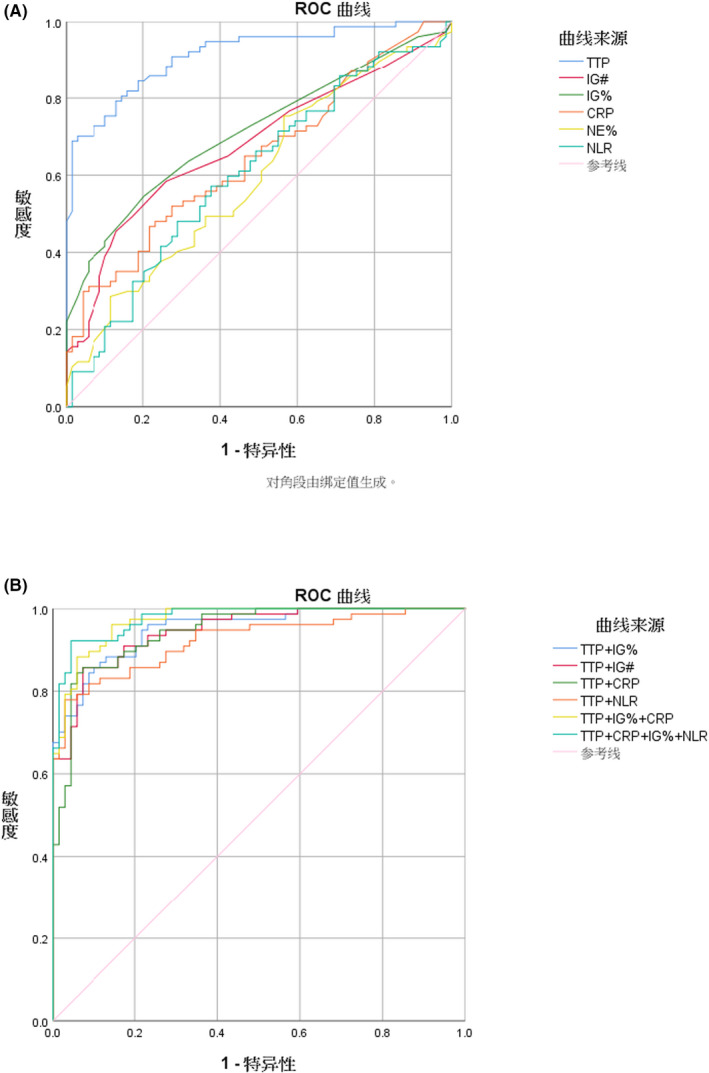
ROC analysis of TTP and inflammatory parameters in identifying CoNS bloodstream infection from contamination. A, ROC curve analysis of single indicators including IG%, IG#, CRP, NE%, and NLR. The AUC of TTP was the highest, reaching a value of 0.913, the AUC of IG% was 0.712, and the AUC of IG#, CRP, NE%, and NLR were 0.679, 0.645, 0.595, and 0.605, respectively, which were all evidently lower than 0.7. B, ROC curve analysis of TTP combined with inflammatory parameters. When TTP was combined with IG%, IG#, CRP, or NLR, the AUC was 0.951, 0946, 0.945, and 0.923, respectively. When TTP was combined with IG%, CRP, and NLR, the AUC was the highest, with a value of up to 0.977

ROC curve analysis was performed on TTP, IG%, IG#, CRP, NE%, and NLR according to the indicators of the statistical differences between the CoNS infection group and the contamination group (Figure 1A). Among the six indices, the TTP area under the curve (AUC) was the highest and reached a value of 0.913 (*P* < .001; Table 3). When the optimal critical value was 23.9 hours, the sensitivity, specificity, positive predictive value, negative predictive value, and accuracy were 0.827, 0.852, 85.2%, 81.3%, and 83.5% (214/256), respectively. The AUC of IG% was 0.712 (*P* < .001), and the optimal critical value was 0.55%. The sensitivity, specificity, positive predictive value, negative predictive value, and accuracy were 0.519, 0.797, 53.9%, 80.6%, and 65.6% (168/256), respectively. The AUC values of IG#, CRP, NE%, and NLR were 0.679, 0.645, 0.595, and 0.605, respectively, which were all lower than 0.7.

**TABLE 3:** Efficacy of TTP and inflammatory parameters in identifying CoNS blood stream infection from contamination

Indicators	Critical value	AUC (95% CI)	Sensitivity	Specificity	PPV (%)	NPV (%)	*P* value	Accuracy (%)
TTP (h)	23.9	0.913 (0.868‐0.959)	0.827	0.852	85.2	81.3	0	83.5
IG%	0.55	0.712 (0.629‐0.795)	0.519	0.797	53.9	80.6	0	65.6
IG# (×10^9^/L)	0.05	0.679 (0.591‐0.766)	0.590	0.750	50.0	83.3	0	60.5
CRP (mg/L)	21.9	0.645 (0.557‐0.734)	0.514	0.822	47.5	79.2	.002	66.0
NE%	67.0	0.595 (0.503‐0.687)	0.284	0.880	60.5	50.6	.048	42.5
NLR	1.33	0.605 (0.513‐0.696)	0.571	0.623	62.3	56.3	.029	59.0

### 3.5 Efficacy of combined indicators to identify CoNS infection from contamination

According to the efficacy evaluation data of a single indicator (Table 3), although the area under the IG#, CRP, and NLR curves was lower than 0.7, considering that IG% and IG# were immature granulocyte parameters, CRP was a common inflammatory index in the laboratory, and NLR was an emerging and readily available inflammation parameter, and then, IG#, CRP, and NLR were included in the joint analysis. By contrast, NE% was not included because the AUC had not attained or exceeded a value of 0.6.

The ROC curve analysis shows (Figure 1B) that TTP combined with IG%, IG#, or CRP can improve the discrimination efficiency of CoNS infection and peripheral toxemia as might be seen in sepsis/septicemia; however, IG% was completely consistent with an improved efficacy of IG# (Table 4). Considering that IG# is susceptible to the WBC count, a combination of inflammatory indicators was not included. When the TTP was combined with IG%, CRP, and NLR, the AUC was 0.977(*P* < .001), and the sensitivity, specificity, positive predictive value, negative predictive value, and accuracy were increased to 0.922, 0.957, 92.2%, 91.3%, and 91.8%, respectively (234/256).

**TABLE 4:** Efficacy of combined indicators to identify CoNS infection from contamination

Combined Indicators	AUC (95% CI)	Sensitivity	Specificity	PPV (%)	NPV (%)	Accuracy (%)
TTP + IG%	0.951 (0.920‐0.982)	0.844	0.913	87.2	84.7	87.5
TTP + IG#	0.946 (0.914‐0.979)	0.857	0.928	88.5	84.7	89.1
TTP + CRP	0.945 (0.911‐0.979)	0.844	0.942	90.0	83.3	89.1
TTP + NLR	0.923 (0.880‐0.966)	0.779	0.971	82.4	84.5	83.3
TTP + CRP ＋ IG%	0.972 (0.951‐0.993)	0.883	0.942	92.2	87.0	91.4
TTP + CRP ＋ IG% + IG#	0.972 (0.952‐0.993)	0.883	0.942	92.2	87.0	91.4
TTP + CRP ＋ IG% + NLR	0.977 (0.959‐0.996)	0.922	0.957	92.2	91.3	91.8

## 4 DISCUSSION

With the widespread use of immunosuppressants, broad‐spectrum antibiotics, and the increasing number of interventional treatments, CoNS has gradually emerged as one of the most commonly isolated bacteria by blood culture.^6^ However, coagulase‐negative *Staphylococcus*, as normal bacteria on the skin surface, can quite readily cross‐contaminate the blood samples, which can then be injected into the blood culture bottle when drawing blood, thus misleading the clinical use of antibiotics.

XuYaping et al reported that the contamination rate of CoNS was 44.76% (64/143). In this study, the statistical analysis of CoNS that were isolated from our hospital in 2019 found that the contamination rate was 48.4% (124/256). Among the CoNS ratio, *Staphylococcus hominis* and *Staphylococcus epidermidis* were the most easily contaminated bacteria in clinical practice, and *Staphylococcus capitis* and *Staphylococcus haemolyticus* also have a certain proportion, which is consistent with the literature.^7^ Since blood culture contamination cannot be avoided completely, it is necessary to identify CoNS bloodstream infections from that of inadvertent cross‐contamination in clinical work practices.

Up to now, many studies in the literature have reported that TTP has a certain predictive value for the clinical significance of CoNS in blood culture,^2^, ^3^, ^8^ which is also confirmed by the results of this current study by our group. Zhang Yu et al studied 160 children and found that the optimal critical value for TTP that could be used to determine a child's blood flow infection and contamination was 17.35 hours. In this study, the optimal critical value of TTP was 23.9 hours and the accuracy was 83.5%. GuoJianlian et al reported that in the study of 137 cases of positive blood culture specimens, the median TTP of the CoNS infection group was 16.42 hours, while that of the contamination group was 38.18 hours. Studies from Ireland showed that the TTP of all pathogens of bloodstream infections was <24 hours.^8^ A more recent study by Pan et al, gave a TTP of 22.72 hours.^2^ American Pardo believed that there was almost no real blood stream infection detected after 48 hours of culture, so it was recommended that antibiotic use should be downgraded after 48 hours, which was also verified in the study of Abdelhamid.^6^, ^9^ According to Vamsi et al, it was found that if the blood culture was negative at 36 hours, there was a 99.14% probability that it would remain negative.^10^ This may be related to differences in case selection in different regions (such as the inclusion criteria of age and disease‐causing bacterial species) and the judgment criteria of bloodstream infections. Previously, it was believed that TTP was associated with an initial bacterial concentration in the blood of patients. The inoculation volume of children was less than that of adults. In addition, a considerable number of children may have received antibiotics before admission. Consequently, the TTP time of children may be longer than that of adults. However, our results indicated that the TTP of CoNS infection in children was essentially consistent with that in adults. Generally, a positive alarm within 24 hours might formally indicate the possibility of a blood flow infection, while more than 1 day can be considered as pollution.

Recent studies have found that peripheral blood IG not only plays an important role in the diagnosis of blood diseases, but also in the occurrence and development of inflammation and infectious diseases. Foreign scholars have found that under conditions where the body is accompanied by infection or systemic inflammation, then the number of peripheral blood circulating immature granulocytes was significantly increased.^11^ However, the optimal threshold for evaluating bloodstream infection and contamination varies from one study to another (0.3%‐0.55%).^12^, ^13^ Latvian scholar Pavare found that an increase of IG% in children was related to evidence of a bloodstream infection.^14^ In this study, both IG% and IG# in children that presented with *Staphylococcus aureus*and CoNS bloodstream infections were higher than normal; however, IG% was slightly better than that of IG# in the identification of CoNS infection and contamination.

In addition, in this study, it was found for the first time that IG% was the highest for diagnosing CoNS among the single inflammatory indicators. Taking 0.55% as the critical value, the sensitivity was 0.519, the specificity was 0.797, and the accuracy attained a value of 65.6%, all of which were higher than similar parameters reported for white blood cells and even CRP.

In our analysis, IG might be more closely related to systemic infection such as sepsis, while other inflammatory indicators will be increased due to local inflammation. Thus, the results of IG% in infected patients can assist in determining whether blood culture CoNS positivity is caused by an infection or from inadvertent cross‐contamination.

Parameters that included WBC, NE%, and CRP are commonly used inflammatory indicators in clinical practice, but the diagnostic value of WBC for neonatal sepsis is relatively low,^15^ while CRP is also highly sensitive to acute infection and tissue injury that might actually be normal under conditions of local and chronic low‐grade infection. Thus, an adjudication of bloodstream infection has certain limitations.^16^ Some scholars even believe that the differential efficacy of IG% on bloodstream infection is equivalent to that of WBC combined with CRP.^17^

There is accruing evidence that neutrophil‐to‐lymphocyte ratio (NLR) is an easy‐to‐obtain inflammatory parameter and results a reliable predictor in a variety of disease conditions characterized by the involvement of immune cells and inflammatory processes^18^, ^19^. When the patient's condition is serious, especially when the total number of white blood cells does not increase, the detection of the ratio is more meaningful than the white blood cell count. The results of this study showed that the NLR value of the CoNS infection group is higher than the contamination group, which can effectively identify bloodstream infections, and the identification efficiency was better than WBC and NE%.

The results of this current study showed that the positive alarm time of children's blood culture, which was identified as coagulase‐negative *Staphylococcus* indicated the possibility of blood flow infection within 24 hours, and the accuracy of distinguishing CoNS infection and contamination could be improved from 83.5% to 91.8% in combination with IG%, CRP and NLR.

In view of the relatively high contamination rate of CoNS in blood culture, it is suggested that in the clinical microbiology setting, one should include the positive alarm time TTP for the first‐level report scoping of a positive blood culture in order to assist in an effective clinical identification of CoNS. As a new parameter in diagnosing infectious diseases, IG, NLR should be included in the routine blood report parameters in the laboratory with certain conditions, since sound clinical assessment of a positive blood culture CoNS result, will provide quite useful information.

This study has a limited sample size and the retrospective analysis of data collected at a single site may lead to bias. In the future, large‐scale prospective studies should be conducted to obtain reproducible and effective estimates, and to determine the most predictable baseline cutoff points based on the characteristics of baseline patients.

## CONFLICT OF INTEREST

The authors declare that they have no competing interests.

## AUTHOR CONTRIBUTIONS

Lanlan Zeng, Shuhui Wang, and Minchun Lin performed the data analysis, made the picture, and wrote the article. Yaxing Chen, Qiulian Deng, and HuaminZhong conducted blood culture, bacterial identification, and typing. XiaoshanGuang and Shuwen Yao tested the laboratory inflammatory markers. Haiying Liu contributed to the article review and editing of the drafts. All authors read and approved the final draft of the article prior to its submission for external peer review.

## EMPLOYMENT OR LEADERSHIP

None declared.

## HONORARIUM

None declared.

## Data Availability

The datasets generated and/or analyzed during the current study are not publicly available since the medical records and data are confidential and respect or abide by the patient's legal rights to privacy; however, the data are available from the corresponding author on reasonable request under the consent from close relatives of the patient.
